# Rifaximin-mediated gut microbiota regulation modulates the function of microglia and protects against CUMS-induced depression-like behaviors in adolescent rat

**DOI:** 10.1186/s12974-021-02303-y

**Published:** 2021-11-04

**Authors:** Haonan Li, Yujiao Xiang, Zemeng Zhu, Wei Wang, Zhijun Jiang, Mingyue Zhao, Shuyue Cheng, Fang Pan, Dexiang Liu, Roger C. M. Ho, Cyrus S. H. Ho

**Affiliations:** 1 Department of Medical Psychology and Ethics, School of Basic Medicine Sciences, Cheeloo College of Medicine, Shandong University, Jinan, Shandong People’s Republic of China; 2 Cheeloo Hospital, Shandong University, Jinan, People’s Republic of China; 3 Department of Psychological Medicine, Yong Loo Lin School of Medicine, National University of Singapore, Singapore, Singapore; 4 Institute of Health Innovation and Technology (iHealthtech), National University of Singapore, Singapore, Singapore

**Keywords:** Adolescence, Chronic unpredictable mild stress (CUMS), Rifaximin, Gut microbiome, Short chain fatty acid (SCFA), Microglia, Neurogenesis

## Abstract

**Background:**

Chronic unpredictable mild stress (CUMS) can not only lead to depression-like behavior but also change the composition of the gut microbiome. Regulating the gut microbiome can have an antidepressant effect, but the mechanism by which it improves depressive symptoms is not clear. Short-chain fatty acids (SCFAs) are small molecular compounds produced by the fermentation of non-digestible carbohydrates. SFCAs are ubiquitous in intestinal endocrine and immune cells, making them important mediators of gut microbiome-regulated body functions. The balance between the pro- and anti-inflammatory microglia plays an important role in the occurrence and treatment of depression caused by chronic stress. Non-absorbable antibiotic rifaximin can regulate the structure of the gut microbiome. We hypothesized that rifaximin protects against stress-induced inflammation and depression-like behaviors by regulating the abundance of fecal microbial metabolites and the microglial functions.

**Methods:**

We administered 150 mg/kg rifaximin intragastrically to rats exposed to CUMS for 4 weeks and investigated the composition of the fecal microbiome, the content of short-chain fatty acids in the serum and brain, the functional profiles of microglia and hippocampal neurogenesis.

**Results:**

Our results show that rifaximin ameliorated depressive-like behavior induced by CUMS, as reflected by sucrose preference, the open field test and the Morris water maze. Rifaximin increased the relative abundance of Ruminococcaceae and Lachnospiraceae, which were significantly positively correlated with the high level of butyrate in the brain. Rifaximin increased the content of anti-inflammatory factors released by microglia, and prevented the neurogenic abnormalities caused by CUMS.

**Conclusions:**

These results suggest that rifaximin can regulate the inflammatory function of microglia and play a protective role in pubertal neurodevelopment during CUMS by regulating the gut microbiome and short-chain fatty acids.

**Supplementary Information:**

The online version contains supplementary material available at 10.1186/s12974-021-02303-y.

## Introduction

Depression is a pervasive neuropsychiatric disorder that is characterized by affective flattening, slow mental processing and hypobulia [1]. The incidence of depression increases sharply during adolescence [2]. Although it is currently recognized that the pathogenesis of adolescent depression involves neurodevelopmental, genetic psychosocial domains, the pathophysiological mechanisms of adolescent depression have not been fully elucidated [3]. Accumulating evidence on the gut microbiome has highlighted the potential link between structural changes in the fecal microbiome and the development of depression, and researchers have proposed a microbial–gut–brain axis to explain the relationship between the microbiome and the brain [4–6]. Furthermore, the role of microglia in the neuroinflammatory response that occurs in the development of depression has been demonstrated [7, 8]. Microglia displays distinct morphologies as either amoeboid or ramified cells, indicating distinct functional states. After stimulation by external pathogenic factors, such as lipopolysaccharide (LPS) and interferon-γ (IFN-γ), microglia transform into the amoeboid-state and secrete a large number of inflammatory mediators and cytotoxic molecules, leading to damage in peripheral nerve cells [9, 10]. Conversely, ramified-state microglia play a neuroprotective role by secreting anti-inflammatory factors, such as Interleukin-10(IL-10) [11]. Therefore, regulation of gut brain axis and microglial function may be a new strategy for the treatment of depression.

The gut–brain axis involves two-way interactions between the gut microbiota and the central nervous system. Different signals from the gastrointestinal tract can regulate brain function through neural, endocrine, immune, metabolic and other pathways [12]. Importantly, changes in gut microbiota can modify their biochemical interactions with the central nervous system as a result of their altered molecular outputs, especially microbial-related metabolites [13]. Although the biological mediators that drive these effects remain largely unknown, methods that change the structure of the gut microbiome have been shown to affect the stress response, emotional and cognitive processes and behavior of animals [14, 15].

Short-chain fatty acids (SCFAs) are microbial metabolites that constitute the main bacterial fermentation products of dietary fiber in the gut. These molecules are generally considered to be key candidate factors in microbiota–gut–brain communication due to their ability to easily cross the blood–brain barrier. Cumulating evidence is gradually clarifying their potential value in immune regulation [16, 17]. An SCFA mixture reduces the secretion of cytotoxins by stimulating THP-1 microglia-like cells, and the use of formate alone reduces the production of reactive oxygen species microglial cells [18]. However, few studies have directly explored the role of SCFAs as potential mediators to affect the microbiota­targeted interventions in affective and cognitive functioning.

Adolescence is a developmental period characterized by significant brain maturation [19]. The heightened neural plasticity of adolescence makes the brain particularly vulnerable to the environmental influences, especially stress. Stress events may increase vulnerability to psychiatric illness by altering the developmental processes of brain regions related to affective and cognitive processing [20]. Hippocampal neurogenesis starts with the proliferation of neural progenitor cells (NPCs) located in the subgranular zone (SGZ) of the hippocampal dentate gyrus (DG), which is associated with the modulation of cognitive processes, such as pattern separation and cognitive flexibility [21, 22]. The dysregulation of hippocampal neurogenesis leads to memory and learning deficits and has been associated with the onset of depression and anxiety disorders [23]. Neurons communicate with each other through synapses located on dendritic spines [24]. Long-term chronic stress inhibits the production of new neurons in the hippocampus and reduces the density of dendritic spines, leading to memory impairment [25, 26]. The number and shape of dendritic spines play important roles in synaptic plasticity, learning and memory.

Traditional antibiotics, such as Beta-lactamics and fluoro-quinolones, in addition to their bactericidal or bacteriostatic functions, often have a profound impact on the composition of gut microbiome, mainly manifested in the reduction of probiotics, such as Bifidobacteria, and the increase of potential pathogenic bacteria, such as Enterobacteriaceae [27]. Rifaximin is a non-absorbable antibiotic that has a high safety profile due to its low systemic absorption. Rifaximin has been shown to inhibit bacterial overgrowth in the small intestine and prevent recurrent hepatic encephalopathy [28]. Rifaximin has important antibacterial and anti-inflammatory effects in the colon, including reduced bacterial virulence and translocation. In addition, rifaximin seems to have a unique prebiotic properties in promoting the growth of beneficial bacteria, such as Bifidobacteria and Lactobacilli, thereby actively regulating the composition of the gut microbiome [29, 30]. Animal models and metagenomic analysis showed that, different from the reduction of flora diversity caused by traditional antibiotics, rifaximin keeping stable the overall composition of on gut microbiome diversity [31]. After the withdrawal of rifaximin, the changes in the gut microbiome composition were stable for a long time [32]. Rifaximin also showed a potential modulate on bacterial metabolic function [33].Therefore, study on the regulatory effect of rifaximin on gut microbiome will help to promote the understanding of gut–brain axis.

Given the evidence that the gut–brain axis and neuroinflammation are involved in depression, we hypothesized that rifaximin has a microbial-dependent antidepressant effect that involves the regulation of microglial function and neurogenesis. We investigated the effects of chronic rifaximin administration on microglial functions and hippocampal neurogenesis in an adolescent rat model of depression. Furthermore, to explore how gut microbiome affect the activation of microglia, we assessed the relation between SCFAs and microbiome. Finally, we investigated the effect of sodium butyrate on the microglia LPS response.

## Materials and methods

### Animals and drug administration

Male Sprague Dawley rats (*n* = 56, 3 weeks old) were purchased from the experimental animal center of Shandong university (Jinan, China). All rats were housed under standard environmental conditions (22 ± 0.5 °C, 50% ± 5% humidity, and a 12 h light/12 h dark cycle) and maintained with free access to a standard laboratory pellet diet and water. The rats were habituated to their new environment for a week, and changes in their sucrose preference, body weight and sucrose consumption before and after drug administration were recorded. The weights and sucrose preferences in the first week were used as the baseline. After their acclimatization, the rats were divided randomly into four groups: control group (CON, *n* = 14), rifaximin group (CON + R, *n* = 14), CUMS model group (CUMS, *n* = 14) and CUMS-exposed rats treated with rifaximin (CUMS + R, *n* = 14).

#### Chronic unpredictable mild stress procedure

The CUMS procedure was performed as previously described [34] with a slight modification. Rats in the CUMS and CUMS + R groups were randomly exposed to different stressors: cage tilting for 24 h, cold swimming for 3 min (at 0 °C), water or food deprivation for 24 h, level shaking for 15 min, tail nip for 1 min (1 cm from the end of the tail), 45 °C heat stress for 5 min and inversion of the light/dark cycle for 24 h. These stressors were applied for 28 days, during which each stressor was applied 4 times. The rats were exposed to different stressors at random every day, making it impossible for the animals to predict the stimulus. The same stressor was not applied on consecutive days. The CON and CON + R group were undisturbed except for necessary procedures. Feces were collected on the last day of the CUMS procedure.

#### Pharmacological treatments

Rifaximin was purchased from Yuanye Bio-Technology Co. Ltd (Shanghai, China). Rifaximin was mixed with normal saline 0.9% in high-speed vortex state. During the 4 weeks of CUMS procedures, rats in CUMS + R and CON + R groups were treated with rifaximin (150 mg/kg) by oral gavage once daily (at 16:00) for 4 weeks. The doses of rifaximin were chosen based on a previous study [35]. The procedures were approved by the Institutional Animal Care and Committee, Shandong University.

### Behavioral tests

#### Sucrose preference test (SPT)

The sucrose preference test (SPT) was administered to quantify loss of interest in rewarding stimuli. Baseline measurements were taken during adaptation, during which rats were placed in different cages and exposed continuously to two bottles for 12 h, one containing sucrose water (1% (wt/vol)) and one containing tap water (normal water). Rats (*n* = 14 per groups) were deprived of water and food for 12 h before the preference test. During the preference test, rats were housed in individual cages followed by free access to 2 bottles of fluids containing a sucrose solution (1% sucrose, 200 mL) and water (200 mL), respectively, for 12 h. The placement of the two bottles was changed after 6 h to prevent the possible effects of preference for a side on drinking behaviors. The consumption of the sucrose solution and tap water was calculated by weighing the bottle. The sucrose preference (SP) value was calculated as follows: sucrose intake (g) × 100%/[sucrose intake (g) + water intake (g)].

#### Open field test (OFT)

The OFT was used to assess exploratory activities and anxiety-like behavior in an open box. The open device was a square acrylic box (150 × 150 × 50 cm^3^) divided into 25 squares at the bottom. All of the rats were placed in the center of the open field apparatus and allowed to explore freely for 5 min. The number of squares crossed (four paws placed on a new square) and the time spent in the center area were recorded by video tracking software (SMART 2.5, Spain). After each rat was tested, the site was swabbed with a wet cloth and dried with a hot air blower.

#### Morris water maze (MWM)

Spatial learning and memory were assessed using the Morris water maze. Rats (*n* = 14 per groups) were trained continuously for 5 days with four quadrants, and the order of the quadrants in each experiment was changed randomly. The platform was hidden 1 cm below the surface of the water and was located in the center of a quarter of the pool. The rats were directed to the platform if they could not locate it within 90 s. After reaching the platform, the rats remained for 10 s before being removed. After each experiment, the rats were dried with cotton wool, placed in cages and kept warm in a room at a constant temperature of 28 °C. Spatial memory was tested on the sixth day with the platform removed. The escape latency time (time taken to find the platform) and the percentage of time spent and entry frequency in the target quadrant (the quadrant containing the platform) were recorded. Performance was recorded by video tracking software (SMART 2.5, Spain).

### 16S rRNA analysis of fecal microbiota

The experiments included extracting the total DNA from samples (*n* = 5 per groups) of the faeces. The data were analyzed on the free online Majorbio I-Sanger Cloud Platform. Total DNA was extracted according to the instructions of the E.Z.N.A.® SOIL Kit (Omega Bio-Tek, Norcross, GA, U.S.). The concentration and purity of DNA were measured using a NanoDrop 2000 spectrophotometer, and the quality of the DNA extraction was confirmed by 1% agarose gel electrophoresis. PCR amplification of the V3–V4 variable region was performed using 338F (5′-ACTCCTACGGGAGCAGCAG-3′) and 806R (5′-GGACTACHVGGGTWTCTAAT-3′) primers. The microbial composition was analyzed via 16S rRNA sequencing by Shanghai Majorbio Bio-pharm Technology (Shanghai, China) according to standard instructions.

### SCFA concentration analysis

The concentrations of SCFAs (acetic acid, propionic acid, isobutyric acid, butyric acid, isovaleric acid, valeric acid and hexanoic acid) in the serum and brain (n = 4 per groups) were determined on a Thermo TRACE 1310-ISQ system (Thermo, USA) fitted with an Agilent HP-INNOWAX column (30 mm × 0.25 mm × 0.25 µm, Agilent, United States). Standard solutions of acetic acid, propionic acid, butyric acid, isobutyric acid, valeric acid, isovaleric acid and hexanoic acid were diluted with ethyl ether to 0.02 μg/mL, 0.1 μg/mL, 0.5 μg/mL, 2 μg/mL, 10 μg/mL, 25 μg/mL, 50 μg/mL, 100 μg/mL, 250 μg/mL and 500 μg/mL, respectively. One hundred microliters of 15% phosphoric acid was added to each 200 μL serum sample or 50 μg of brain tissue, and then 20 μL of 75 μg/mL isohexanoic acid solution and 280 μL of diethyl ether were added. The samples were centrifuged at 4 °C and 12,000 rpm for 10 min. The supernate was taken for testing. The mixture was stored at − 20 °C.

### Histopathological examination

The colons were soaked in 4% paraformaldehyde in 0.1 mol/L PBS (pH 7.4) for 24 h, dehydrated and embedded in paraffin, cut into 5 μm slices transversely with a microtome, stained with hematoxylin and eosin (HE) and analyzed by Olympus BX53 microscope.

### Immunofluorescence

Rats (*n* = 4 per groups) were deeply anesthetized with 10% pentobarbital (10 mL/kg) and slowly perfused with 300 mL of phosphate buffer saline (PBS, pH 7.2), followed by 300 mL of 4% paraformaldehyde (PFA). The brain was removed and immobilized in 4% PFA for 72 h and then dehydrated in 30% sucrose for 72 h. Tissues on slides were treated with 0.2% Triton X-100 and blocked with 5% goat serum. The slide was incubated overnight at 4 °C with the following primary antibodies: Ki-67 (D3B5) Rabbit mAb (Ki-67, 9129, Cell Signaling); Anti-Doublecortin (DCX, ab254133, Abcam); Anti-Iba1 antibody (Iba-1, ab178846, Abcam); Anti-PSD95 antibody (ab238135, Abcam); Anti-CD68 antibody (ab125212, Abcam), Neuronal nuclei (E4M5P) Mouse mAb (NeuN, 94403, Cell Signaling). Anti-ZO1 tight junction protein antibody (ZO-1, ab221547, Abcam) and Anti-Claudin 1 antibody (Claudin-1, ab211737, Abcam). After the primary antibodies were rinsed with PBS, the tissues were covered with Alexa Fluor® 488- and Alexa Fluor® 594-conjugated fluorescent secondary antibodies and incubated in the dark for 60 min. After rinsing the secondary antibodies with PBS, 4,6-Diamidino-2-phenylindole (DAPI)solution was added, and the slides were incubated in the dark for 3–5 min. The slides were observed with an Olympus BX53 fluorescent microscope equipped with a DP74 Microscope Digital Camera.

### Golgi staining

After the rats (*n* = 4 per groups) were sacrificed and perfused with normal saline, the brain was removed and immediately fixed in 30% sucrose solution for 48 h. Then, the brain was treated with a Golgi staining kit (g1069, Servicebio, China) for 5 days. After staining, tissue blocks were cut into 50-µm-thick sections, and then sections were dehydrated in absolute ethanol twice for 20 min and cleared in xylene for 30 min. The images were obtained using a VS120-S6-W system (OLYMPUS, Japan) and analyzed with Olympia ver. 2.9.

### Enzyme-linked immunosorbent assay (ELISA)

The concentrations of Interleukin-1ra(IL-1ra), IL-10, Tumor necrosis factor-α(TNF-α) and Interleukin-1ra (IL-1β) in the brain tissue(*n* = 4 per groups), peripheral serotonin and glucocorticoid, supernatant and cell cultures were measured with validated specific ELISA assays according to the manufacturer’s instructions (Elabscience, Wuhan, China). Each sample (100 μL) was added to ELISA plates in triplicate. A reference standard was used to establish the working curve, and the biotinylated antibody, HRP conjugate solution, substrate reagent and terminal solution were successively added according to the instructions. The inflammatory cytokine levels were measured by a microplate reader at 450 nm absorbance.

### Primary microglia culture and treatment

The brain tissue of newborn (P0–P5) SD rats was extracted for primary microglia culture. The brain tissue was enzymatically dissociated into single cell suspension with 0.25% trypsin and filtered with 70 µm cell filter. Then, mixed glial cells were cultured in high-glucose Dulbecco’s modified Eagle’s medium (Gibco, Thermo Fisher Scientific) containing 10% fetal bovine serum (Gibco) at 37 ℃ and 5% CO_2_ for 1 week. Microglia were mechanically isolated from mixed glial cultures and inoculated into 24-well plates at a density of 1 × 10^5^ cells/cm with a humidified atmosphere of 95% air and 5% CO_2_. Lipopolysaccharide (LPS; 100 ng/mL, Sigma) was used to activate microglial cells. The cells were treated with different concentrations of sodium butyrate (SB, MedChemExpress): 0.1 μM, 0.3 μM and 0.5 μM. Then, the in vitro cultured microglia were divided into following groups: CON, LPS (treated with LPS for 24 h), SB (treated with SB for 24 h) and LPS + SB (treated with LPS and SB for 24 h). After the indicated treatments, the cells were used to assess the microglial function.

#### Flow cytometry analyses

Cells were incubated with fluorescently labeled APC Anti-CD68 antibody (ab283654, Abcam) (1:100) for 30 min. FACS was performed with the BD FACS Aria II cytometer (Becton Dickinson), and the data were analyzed using Kaluza Analysis 2.1 (Beckman Coulter Life Sciences, USA).

### Statistical analysis

All experimental data are represented as the mean ± SEM. Except for the Morris water maze (MWM) test results, data were analyzed by two-way analysis of variance (ANOVA), followed by Tukey’s multiple comparison tests. Repeated-measures two-way ANOVA followed by post hoc Bonferroni multiple comparison tests were used for the Morris water maze. Principal coordinates analysis were generated by QIIME1.8 software to examine sample clusters. The Spearman correlation test was used to analyze the correlation between the gut microbiome and short-chain fatty acids, the Spearman test and the corresponding *P* values were calculated using the cor.test() function with two-sided alternative hypothesis, and *P* values were corrected for multiple comparisons using the *q* value package in R. Parameters were analyzed using GraphPad Prism 8 (GraphPad Software, USA). The analysis results were only further evaluated when a significant difference was observed. *P* ˂ 0.05 or *q* ˂ 0.1 was considered statistically significant.

## Results

### Rifaximin ameliorated CUMS-induced depressive-like behaviors

**Fig. 1:**
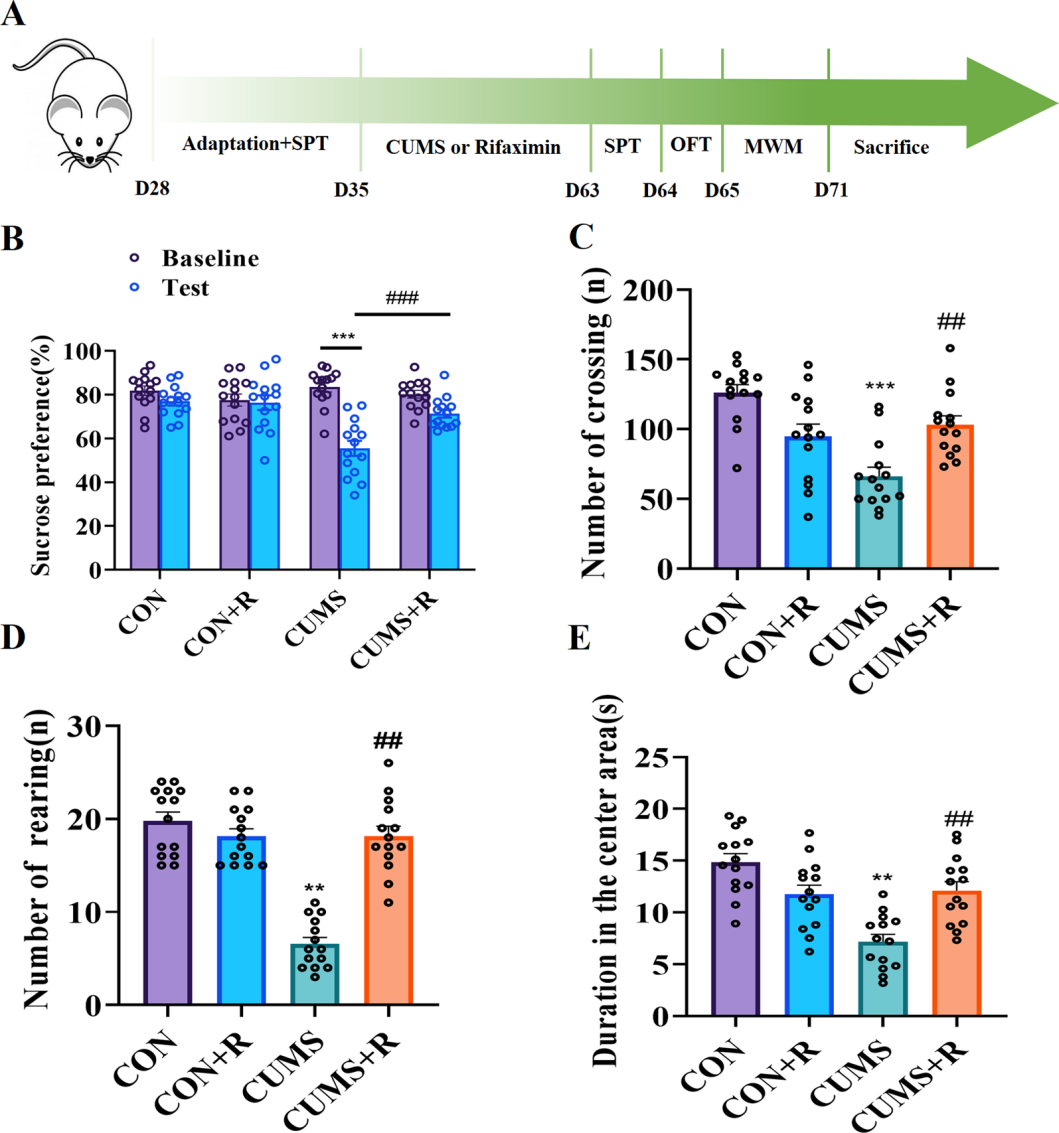
Depressive-like behavior induced by CUMS and the protective effect of rifaximin. **a** Experimental flowchart. **b** Sucrose preference. **c** Frequency of crossing. **d** Number of rearing. **e** Duration in the center area. *P < 0.05, **P < 0.01, ***P < 0.001 vs. the CON group; ^#^*P* < 0.05, ^##^*P* < 0.01, ^###^*P* < 0.001 vs. the CUMS group

The experiment was designed according to the steps in Fig. 1a. The SPT was performed individually to lessen the effect of variability in sucrose preference. Low sucrose intake reflects an impaired reward response and anhedonia, one of the core symptoms of major depression. The CUMS-treated rats showed significantly decreased sucrose consumption (*F* (3, 39) = 12.43, *P* < 0.001), and this effect was prevented in CUMS-exposed rats that received rifaximin (*P* = 0.0075) in Fig. 1B. The OFT was used to measure movement and exploratory behavior in new environments. The CUMS treatment resulted in a decrease in the total frequency of crossing (*F* (3, 39) = 14.72, *P* < 0.001)and rearing (*F* (3, 39) = 14.97, *P* = 0.0019) compared with the CON group (Fig. 1c, d), and a marked decrease in the time spent in the central area (*F*(3,39) = 12.83, *P* = 0.0063)was observed in the CUMS-exposed rats when compared with the CON group(Fig. 1e). These decreases in spontaneous movement (crossing, rearing and center duration) were not present in CUMS + R rats. The learning ability and spatial memory of rats were tested using the MWM. On days 3 and 4, CUMS-exposed rats had a longer escape latency than the CON (*F*(3, 380) = 12.83, D3: *P* < 0.001; D4: *P* = 0.004) and CUMS + R (*P* = 0.0479) groups (Additional file 1: Fig. S1A). After removing the platform, CUMS-exposed rats spent less time in the target quadrant (*F*(3,39) = 12.4, *P* < 0.001) and crossed the platform fewer times (*F*(3,39) = 20.37, *P* < 0.001) than the CON group (Additional file 1: Fig. S1B, C). These results indicate that CUMS impaired learning and spatial memory. In sharp contrast, rats in the CUMS + R group spent more time in the target zone (*P* = 0.0046) and crossed it more frequently (*P* < 0.001) in the search session compared with the CUMS-exposed rats, showing a circular motion centered around the platform area(Additional file 1: Fig. S1D). However, rifaximin treatment alone had no significant effect on learning and spatial memory compared with the CON group.

### Rifaximin modulates the microbial composition of CUMS rats

**Fig. 2:**
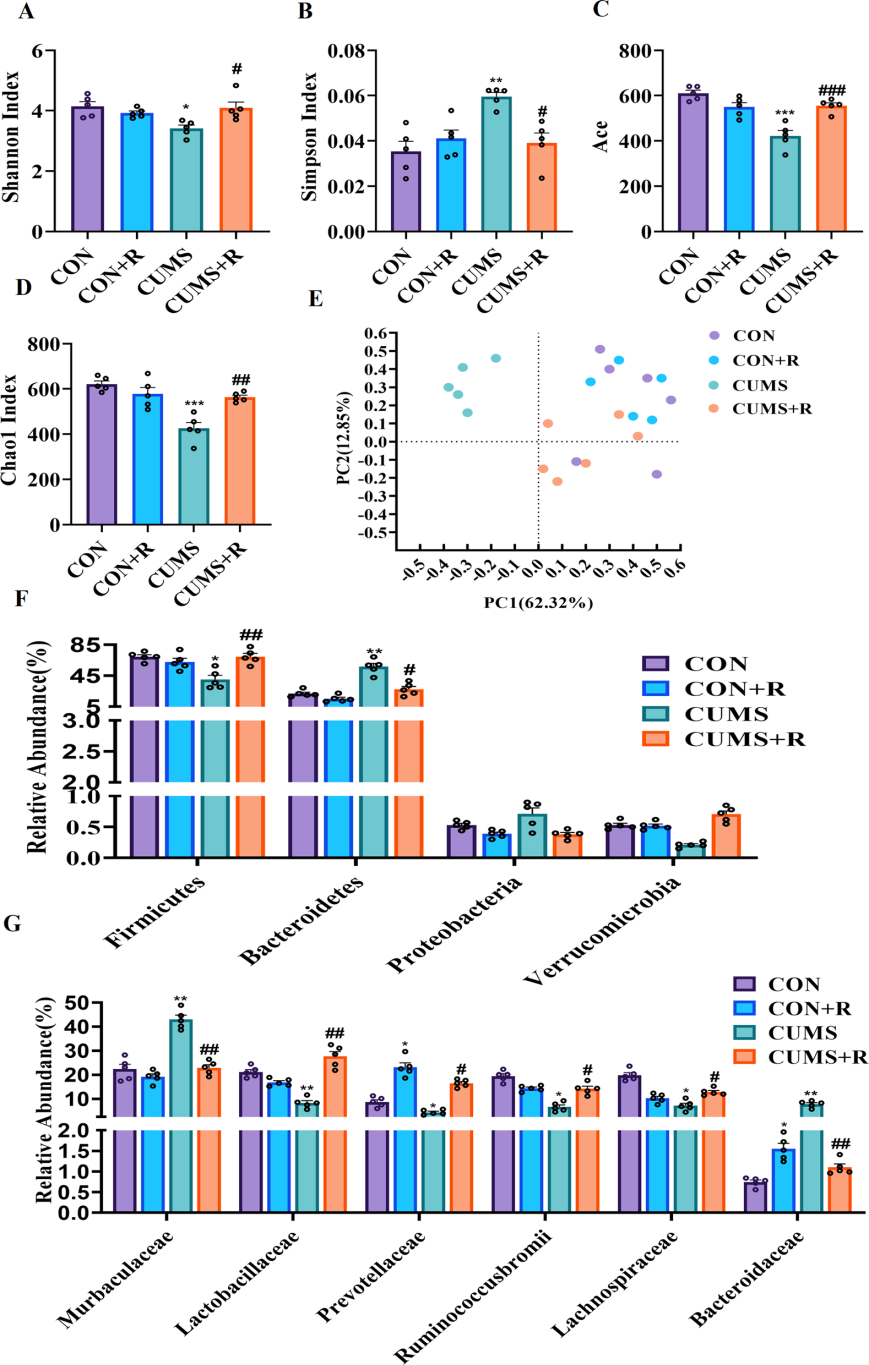
Effects of rifaximin on the microbial composition and integrity of intestinal mucosa of CUMS rats. **a** Shannon index. **b** Simpson index. **c** Ace index. **d** Chao1 index. **e** Principal coordinate analysis. **f** Relative abundance of distinguishable phyla. **g** Relative abundance of distinguishable family. *P < 0.05, **P < 0.01, ***P < 0.001 vs. the CON group; ^#^*P* < 0.05, ^##^*P* < 0.01, ^###^*P* < 0.001 vs. the CUMS group. q values were verified by the Benjamini and Hochberg correction post hoc test

Previous studies have shown that gut microbiome alterations can affect depressive-like behaviors. Therefore, we attempted to determine whether CUMS-exposed rats exhibited alterations in the gut microbiome. Alpha diversity was analyzed by calculating the Shannon, Simpson, Chao1 and Ace indices on OTU level. The results show that the diversity of the fecal microbiome was reduced in rats in the CUMS group, as Shannon values (*F* (3, 12) = 4.488, *P* = 0.0284) was significantly decreased, while Simpson values (*F* (3, 12) = 7.689, *P* = 0.0041) were significantly increased (Fig. 2a, b). Furthermore, the Ace (*F* (3, 12) = 24.64, *P* < 0.001) and Chao1 (*F* (3, 12) = 19.58, *P* < 0.001) indices of CUMS rats were significantly decreased compared with those of the CON group (Fig. 2c, d), indicating that CUMS reduced the richness of the fecal microbiome. Rifaximin protected against these adverse effects and did not negatively affect the richness of the gut microbiome of CON + R rats.

Beta diversity was analyzed through principal coordinate analysis (PCoA) plots using nonphylogenetic Bray–Curtis metrics to assess differences in microbial composition (OTU) between the four groups (Fig. 2e). There was a clear separation between the CUMS and CON groups along the first principal component (PC1) axis (Adonis *P* value = 0.001), suggesting that CUMS-induced depression can change the structure of the microbiome.

To identify significant changes in the gut microbiome, taxonomic comparisons at the family level were performed for all groups at *q* value < 0.05(Benjamini–Hochberg Correction). At the phylum level, we found that the richness of Firmicutes in the CUMS group (*q* value = 0.0251) was significantly lower than that in the CON group, while the richness of Bacteroides (*q* value = 0.0066) was significantly higher. Rifaximin prevented the change of Firmicutes (*q* value = 0.0041) and Bacteroidetes (*q* value = 0.0382) in CUMS + R group (Fig. 2f). At the family level, total of six families differed in relative abundance from their levels in the CON group (Fig. 2g). Four of the six bacterial families, namely, Lactobacillaceae (Firmicutes, *q* value = 0.0086), Prevotellaceae (Firmicutes, *q* value = 0.047), Ruminococcaceae (Firmicutes, *q* value = 0.035) and Lachnospiraceae (Firmicutes, *q* value = 0.028) were decreased in the CUMS group, and two families, Muribaculaceae (Bacteroidetes, *q* value = 0.0027) and Bacteroidaceae (Bacteroidetes, *q* value = 0.0014) were increased. These results demonstrate that the relative abundances of predominant Firmicutes genera were decreased in CUMS subjects, while those of Bacteroidetes were increased, suggesting that the balance between Firmicutes and Bacteroidetes may be involved in the pathogenesis of depression. Rifaximin increased the richness of Bacteroidaceae (*q* value = 0.022) and Prevotellaceae (*q* value = 0.015) in rats in the CON + R group, and no negative behavioral effects were observed. Next we attempted to determine the effects of CUMS or rifaximin treatment on the integrity of intestinal mucosa. The results of hematoxylin–eosin staining (HE) indicated that there was no significant pathological change in colon (Additional file 2: Fig. S2A). However, the integrity of intestinal mucosal barrier was impaired as there was significantly decreased (*F* (3, 21) = 16.83, *P* = 0.0084) of the median fluorescence intensity(MFI) of zonula occludens-1 (ZO-1) (Additional file 2: Fig. S2B–D).

### The relevance between gut microbes and short-chain fatty acid

**Fig. 3:**
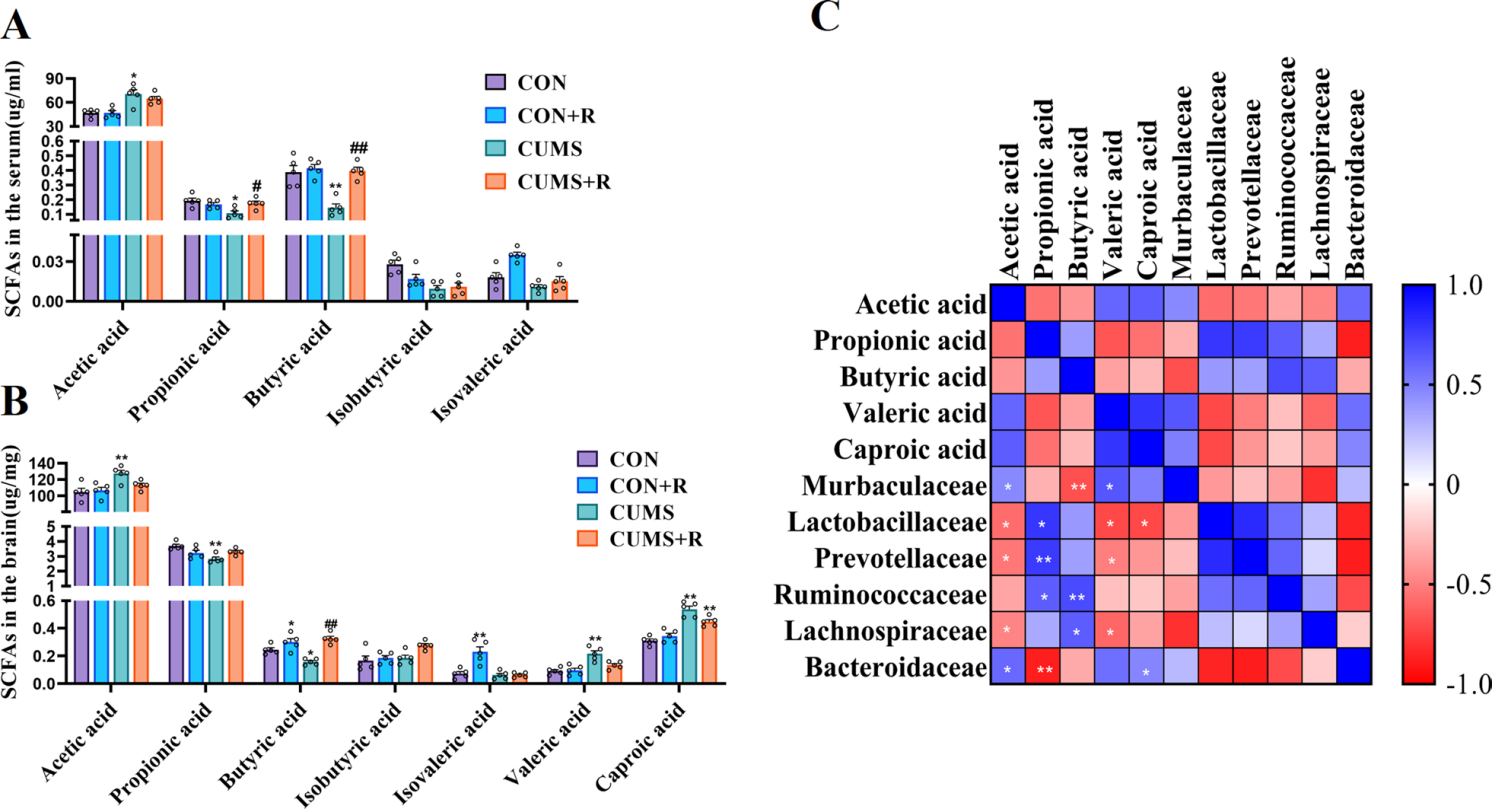
Relevance between the short-chain fatty acid content in the brain and the gut microbiome. **a** Levels of SCFAs in the serum. **b** Levels of SCFAs in the brain. **c** Matrix is used to describe SCFA–microbe correlations. The depth of the color of the square indicates the magnitude of the correlation, where blue squares represent positive correlations and red squares represent negative correlations. White asterisks indicate the significance of the correlation (*q < 0.1, **q < 0.05). In **a** and **b**, black asterisks indicate the significance of the correlation *P < 0.05, **P < 0.01, ***P < 0.001 vs. the CON group; ^#^*P* < 0.05, ^##^*P* < 0.01, ^###^*P* < 0.001 vs. the CUMS group

We analyzed changes in SCFAs caused by the gut microbiome to assess their potential role as key mediators. For this purpose, we measured the SCFA contents (acetic acid, propionic acid, butyric acid, isobutyric acid, valeric acid, isovaleric acid and caproic acid) in the serum and brain by Gas Chromatography–Mass Spectrometer (GC–MS) (Fig. 3a, b). CUMS treatment increased the level of acetic acid (*F*(3, 12) = 11.09, *P* = 0.036) and decreased those of propionic acid (*F* (3, 12) = 13.18, *P* = 0.026) and butyric acid (*F* (3, 12) = 22.09, *P* = 0.0058) in the serum. Rifaximin treatment significantly increased the levels of propionic acid (*P* = 0.041) and butyric acid (*P* = 0.0037) in the serum of the CUMS + R group compared with CUMS rats, whereas it had no significant effect on the content of acetic acid. However, in the brain, rifaximin only upregulated the concentration of butyric acid (*F* (3, 12) = 19.19, *P* = 0.0025). Interestingly, rifaximin treatment increased the butyric acid concentrations not only in CUMS + R group but also in the CON + R group (*P* = 0.015), which indicates that butyrate variation may be a specific function of rifaximin.

To investigate the relationship between microbes and SCFAs after CUMS treatment, we examined correlations between the six microbes enriched in CUMS rats and five SCFAs that varied between groups in the brain (Fig. 3c). Spearman's test revealed 18 significant unique microbe–SCFA correlations in the brain (*q* value < 0.1), with Spearman’s rank correlation coefficient (Spearman *r*) ranging between − 0.88 and 0.76. We present these correlations between microbe abundance and SCFA levels as a matrix. There were two significant positive microbe–SCFA correlations (*q* value < 0.05): Prevotellaceae and propionic acid (Spearman *r* = 0.76, *q* value = 0.032), Ruminococcus bromii and butyric acid (Spearman *r* = 0.71, *q* value = 0.040). There were two significant negative correlations (*q* value < 0.05): Muribaculaceae and butyric acid (Spearman *r* =  − 0.68, *q* value = 0.045), Bacteroidaceae and propionic acid (Spearman *r* =  − 0.88, *q* value = 0.016). These results may reveal part of the mechanism by which rifaximin modulates depressive symptoms through gut microbiota.

We also measured the serotonin and glucocorticoid in plasma. We found that rifaximin and CUMS had no significant effect on serotonin, while rifaximin significantly prevented the increase of glucocorticoid caused by CUMS (Additional file 3: Fig. S3A, B).

### Rifaximin protect against the enhanced pro-inflammatory function and phagocytosis of microglia caused by CUMS

**Fig. 4:**
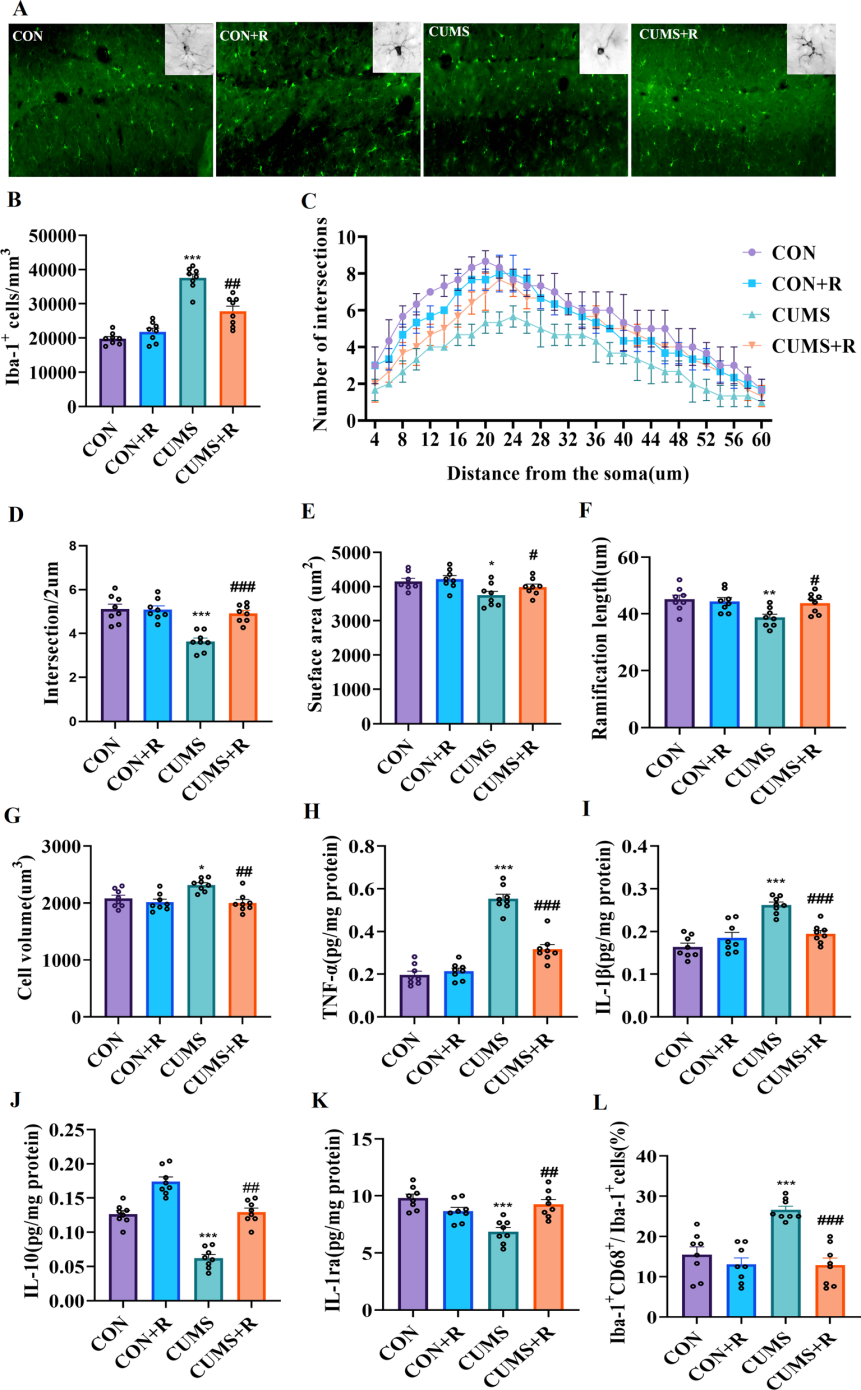
Effects of rifaximin on microglia in the hippocampal DG of CUMS rats. **a** Iba-1 immunoreactivity of microglia in the DG. **b** Total number of microglia. **c** Sholl analysis. **d** Mean number of intersections. **e** Surface area of microglia. **f** Ramification length of microglia. **g** Soma volume of microglia. **h** Level of TNF-α in DG. **i** The level of IL-1β in DG. **j** Level of IL-10 in DG. **k** Level of IL-1ra in DG. **l** Proportion of Iba-1^+^/CD68^+^ cell. *P < 0.05, **P < 0.01, ***P < 0.001 vs. the CON group; ^#^*P* < 0.05, ^##^*P* < 0.01, ^###^*P* < 0.001 vs. the CUMS group

Microglia regulate neuroinflammation in the brain. The results show that the CUMS procedure increased the number of Iba-1^+^ microglia (*F* (3, 21) = 67.16, *P* < 0.001) in the hippocampal DG compared with the CON group. In addition, the number of microglia (Iba-1^+^) was decreased in the CUMS + R group (*P* = 0.003) compared with CUMS group (Fig. 4a, b). To confirm the morphometric changes in microglial cells in response to CUMS in the hippocampal DG, a Sholl analysis of microglial cells was used to characterize the complexity of the cells. CUMS treatment significantly reduced the complexity of microglia: the mean number of intersections (*F* (3, 21) = 16.31, *P* < 0.001) and surface area (*F* (3, 21) = 4.472, *P* = 0.014) were significantly decreased (Fig. 4c–e). Our results also showed the decreased ramification length (*F* (3, 21) = 5.277, *P* = 0.0089) and increased soma volume (*F* (3, 21) = 8.670, *P* = 0.014) in CUMS group (Fig. 4f, g, Additional file 4: Fig. S4A). These morphological alterations are the characteristics of activated microglia. The intersections in the CON and CUMS + R groups reached a peak at 20 µm, while the CUMS group had a peak at 24 µm (Fig. 4c), which indirectly confirms the increased microglia volume. Rifaximin prevented the morphological changes caused by CUMS and kept microglia in a ramified-state.

We further measured the levels of inflammatory cytokines in the hippocampal DG. Rifaximin treatment prevented the CUMS-induced increase of TNF-α (*F* (3, 21) = 72.78, *P* < 0.001) and IL-1β (*F* (3, 21) = 29.70, *P* < 0.001)(Fig. 4h, i), and increased IL-10 (*F* (3, 21) = 54.00, *P* = 0.007) and IL-1ra (*F* (3, 21) = 12.09, *P* = 0.0073) in the DG (Fig. 4j, k). These results confirm the involvement of microglia in CUMS-mediated inflammatory activation and suggest that rifaximin can regulate the inflammatory function of microglia. CD68 is one of the most commonly used markers to describe the phagocytic function of microglia. The proportion of Iba-1^+^/CD68^+^ cell was significantly increased (*F* (3, 21) = 12.81, *P* < 0.001) after CUMS treatment (Fig. 4l, Additional file 3: Fig. S4B), we further found that the microglia of rats in the CUMS group contained more postsynaptic density protein 95 (PSD95), a marker of excitatory glutamatergic synapses on postsynaptic membrane (Additional file 4: Fig. S4C, D), which suggested that microglia have stronger phagocytic activity. Rifaximin treatment normalized the enhancement of phagocytosis induced by CUMS.

Next, we aimed to determine whether microglia are activated by abnormal changes of SCFAs. Based on the unique regulatory effect of rifaximin on cerebral butyric acid content, we hypothesized that the change in microglial function might be related to butyrate. Therefore, we used simple linear regression to analyze the interrelation of brain butyrate content with the expression of microglia-related pro- and anti-inflammatory cytokines. The results show that butyrate content was negatively correlated with TNF-α and IL-1β (Additional file 4: Fig. S4E) and positively correlated with the level of IL-10 and IL-1ra (Additional File 4: Fig. S4F). These results suggest that rifaximin may participate in the regulation of inflammation through butyrate, triggering a change of the microglial function.

### Sodium butyrate induced the functional transformation of microglia in vitro

**Fig. 5:**
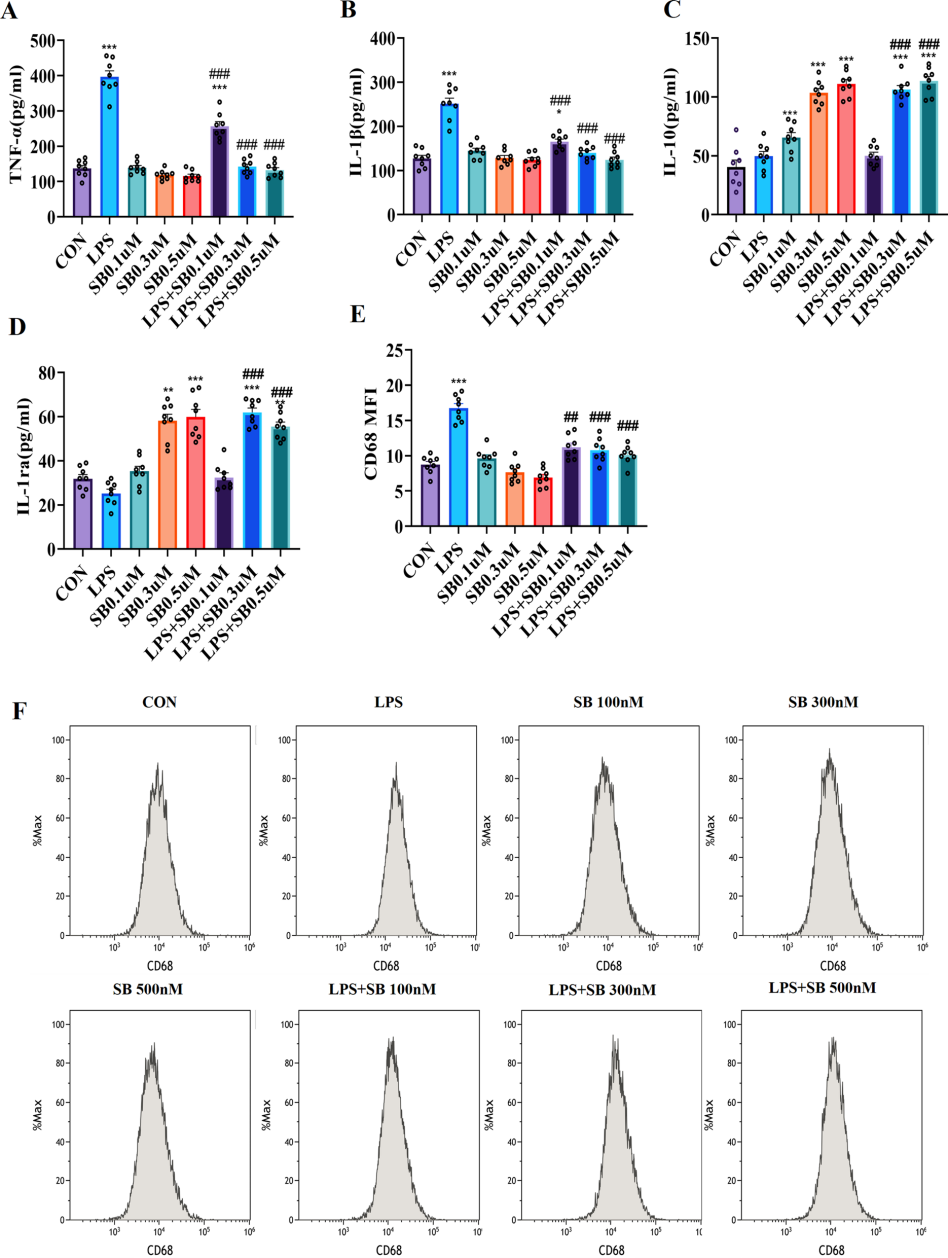
Sodium butyrate regulates the microglia LPS response in vitro. **a** Level of TNF-α. **b** Level of IL-1β. **c** Level of IL-10. **d** Level of IL-1ra. **e** Median fluorescence intensity (MFI) of CD68. **f** Representative FACS plots showing CD68 expression gated on microglia treated with LPS or SB. *P < 0.05, **P < 0.01, ***P < 0.001 vs. the CON group; ^#^*P* < 0.05, ^##^*P* < 0.01, ^###^*P* < 0.001 vs. the LPS group

ELISA were used to assess whether butyrate regulates the inflammatory function of microglia stimulated by LPS or sodium butyrate (SB) in vitro. The GC–MS results showed that the concentration of butyrate in the brain of rats in CUMS + R group is 0.29 ± 0.03uM. We investigated the effects of different doses of SB on microglia (0.1uM, 0.3uM and 0.5uM) on the basis of GC–MS results. We found that SB (0.3uM) suppressed the LPS-induced increase in TNF-α (*F* (7, 49) = 106.4, *P* < 0.001) and IL-1β (*F* (7, 49) = 35.90, *P* < 0.001) (Fig. 5a, b). SB significantly elevated the content of IL-10 (*F* (7, 49) = 71.79, *P* < 0.001) and IL-1ra (*F* (7, 49) = 42.50, *P* < 0.001) in the medium after LPS stimulation (Fig. 5c, d), whereas LPS stimulation had no significant effect on the content of IL-10 and IL-1ra. We also found that SB enhanced the anti-inflammatory function of microglia in a dose-dependent manner. Flow cytometry was performed to study the phagocytosis of microglia (Fig. 5e, f). Increased median fluorescence intensity (MFI) of CD68 were observed in the LPS group (*F*(7, 49) = 35.43, *P* < 0.001), indicating that LPS enhanced the phagocytosis of microglia. The expression of CD68 significantly decreased in LPS + SB 0.3 μM (*P* < 0.001) group compared with LPS group, suggesting that SB alleviate the strong phagocytosis of microglia induced by LPS.

### Rifaximin protected against the neurodevelopmental deficits caused by CUMS

**Fig. 6:**
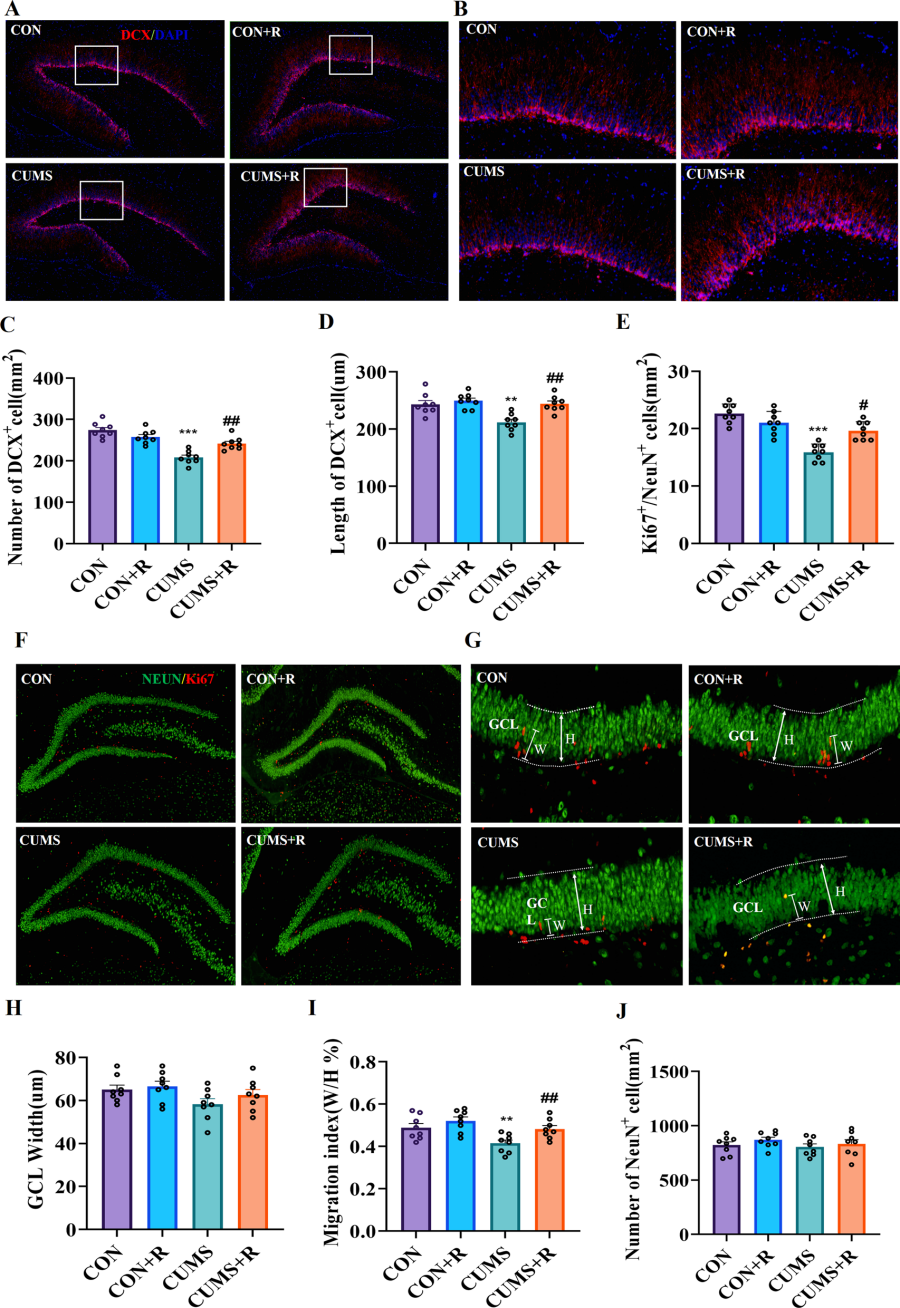
Effects of rifaximin on neurogenesis and synaptic plasticity in the hippocampal DG of CUMS rats. **a** Immunofluorescence for DCX (red) and DAPI (blue) in the DG. **b** Length measurement of DCX^+^ cells. **c** Number of newborn neurons (DCX^+^). **d** Length of DCX^+^ cells. **e** Number of immature neurons (Ki67^+^/NeuN^+^). **f** Immunofluorescence for Ki67 (red) and NeuN (green) in the DG. **g** Immunofluorescence image depicting the migration of immature neurons (Ki67^+^/NeuN^+^). **h** Width of granule cell layer. W: the distance between the center of Ki67^+^/NeuN^+^ cell and the subgranular zone. **h** Width of the GCL. **i** Migration index of immature neurons. **j** Number of mature neurons (NeuN^+^). Values represent the mean ± SEM. *P < 0.05, **P < 0.01, ***P < 0.001 vs. the CON group; ^#^*P* < 0.05, ^##^*P* < 0.01, ^###^*P* < 0.001 vs. the CUMS group

The effect of CUMS on neurogenesis in the hippocampal SGZ is related to two stages of the neurogenic process: differentiation and integration of new cells in the subgranular cell layer of dentate gyrus. To evaluate the differentiation of newborn cells, DCX-a marker of newborn neurons was used to determine the influence of rifaximin on neurogenesis in the hippocampus. As shown in Fig. 6a–d, significant decreases in the number (*F* (3, 21) = 32.47, *P* < 0.001) and length (*F* (3, 21) = 12.19, *P* = 0.0051) of DCX^+^ cells were observed in CUMS rats in comparison with the CON group, suggesting the impairment of neural differentiation. However, rifaximin prevented the negative effect of CUMS on neurogenesis. An outcome of hippocampal neurogenesis is the production of mature dentate granule neurons that migrate and integrate functionally into the granule cell layer (GCL) of DG. Ki67, a marker of proliferation, and NeuN, a marker of mature neurons, were used to trace the integration of immature neurons in DG. The migration index was calculated by the ratio of the distance between the center of Ki67^+^/NeuN^+^ cell and the subgranular zone to the width of granule cell layer. We observed that CUMS led to a significant reduction in the number of immature neurons (*F*(3,21) = 26.31, *P* < 0.001) and significantly altered the migration patterns of immature neurons, as indicated by the lower (*F* (3,21) = 28.14, *P* = 0.0024) migration index(Fig. 6e, f, g, i). Migration defects may alter the integration of immature neurons, since their abnormal position inside the GCL affects their function. We also found that the density of dendritic spines (*F*(3, 21) = 5.470, *P* < 0.001) and the number of mushroom spines (*F* (3, 21) = 7.879, *P* = 0.003) decreased significantly in rats in the CUMS group (Additional file 5: Fig. S5A–C). These results explain the impaired learning ability and memory of CUMS rats in the Morris water maze. Rifaximin protected against the neurodevelopmental deficits. The number of mature neurons was not affected by CUMS or rifaximin (Fig. 6j).

## Discussion

Evidence that the microbiota–gut–brain axis plays a critical role in health and disease, including neuropsychiatric disorders, is rapidly accumulating [36]. These findings suggest that changes in gut microbiome and metabolites play an important role in emotion regulation. Metagenomic studies revealed that the microbiome changed significantly in patients with severe depression [37]. Probiotics has been reported to improve severe depression and intestinal dysfunction in humans [38, 39]. These results suggest that there is a close relationship between the gut microbiome and the pathogenesis of depression, and an understanding of the physiological mechanism underlying these relationships will help in exploring new options for clinical treatment. In this study, using an adolescent rat model of stress-induced depression, we investigated the protective effect of rifaximin on depression-like behavior via alteration of the gut microbiome and analyzed the possible mechanisms. The adolescent rats exposed to chronic unpredictable mild stress (CUMS) exhibited depression-like behaviors, such as decreased sucrose consumption, reduced exploratory behavior and impaired learning and memory ability. The structure of gut microbiome and the levels of short-chain fatty acids in the brains of depressive rats were disturbed by CUMS. Furthermore, CUMS enhanced the pro-inflammatory function of microglia and impaired the neurodevelopment in adolescent rat. We demonstrate that rifaximin enhanced the anti-inflammatory function and prevented the abnormal phagocytosis of microglia by regulating gut microbiome and one of its metabolites, butyrate. Rifaximin also protected against the neurodevelopmental deficits in the hippocampal dentate gyrus. Thus, treatment strategies of regulating gut microbiome in depression patients may ameliorate the behavioral and cognitive changes associated with the disease.

Transplants of the gut microbiome from depressed patients into germ-free mice resulted in depression-like behavior in the recipient mice, suggesting that depressive symptoms can be induced by the alteration of gut microbiome [40].

In the CUMS model, chronic stress not only affects the brain and stress response system but also disturbs the intestinal microbiota. The results of 16S rRNA-based analysis of the gut microbiome showed that the content of Firmicutes of CUMS rats was decreased, while the content of Bacteroides was significantly increased. Haiyin Jiang et al. found that the richness of Bacteroidetes, Proteobacteria and Actinobacteria in the gut microbiome of patients with major depressive disorder (MDD) was significantly increased, while the levels of Firmicutes were significantly decreased [41]. Zhang et al. also stated that the proportion of Firmicutes and Bacteroides is an important factor that distinguishes patients with major depression from healthy people [42], which support our results. Our study further analyzed changes in gut microbiome at family level. We found that the contents of Lactobacillaceae, Prevotellaceae, Ruminococcaceae and Lachnospiraceae in Firmicutes decreased significantly, while those of Muribaculaceae and Bacteroidaceae in Bacteroides increased significantly in CUMS rats. Rifaximin effectively modulated the gut microbial composition and prevented the decrease in Firmicutes and increase in Bacteroidetes caused by CUMS.

Our results showed that there were no significant changes in colonic physiological structure and intestinal mucosal barrier integrity in CUMS rats. Therefore, the gut microbiome may affect the body through its metabolites. Metabolites are important factors in the gut microbiome-mediated regulation of the physiological function of the host. Previous studies have mainly analyzed SCFAs from feces [43]. However, whether fecal SCFAs can be used as a standard to measure the SCFA status of the host is still in question. In this study, we measured the levels of SCFAs in the serum and brain. We found that the content of butyrate in the brain of CUMS rats decreased significantly, while rifaximin prevented decrease of butyrate caused by CUMS. Moreover, rifaximin treatment alone also increased butyrate level in the brain. The correlation between the gut microbiome and brain SCFAs was further analyzed using a Spearman correlation matrix. We found a correlation between several gut microbiota and SCFAs, especially the correlation between Ruminococcus bromii and butyric acid as well as Lachnospiraceae and butyric acid. With the development of metagenomics, we have gained an understanding of different SCFA sources. Ruminococcus bromii and Lachnospiraceae are considered to be among the main sources of intestinal butyrate [44]. Ruminococcus bromii can produce butyrate by fermenting dietary polysaccharides (such as starch and hemicellulose), inulin and pectin derivatives [45]. Lachnospiraceae species have genes for the synthesis of butyrate from acetyl CoA to butyryl CoA, and some genera in this family can also synthesize butyrate from acetic acid and lactic acid [46]. This may explain the regulatory effect of rifaximin on butyrate via the gut microbiome.

Serotonin functions as a key neurotransmitter in the communication system between central nervous system and the gastrointestinal tract [47]. Prebiotics and probiotics in addition to antidepressive effects, have been shown to have a considerable effect on the regulation of serotonin metabolism [48, 49]. However, our results showed that the change of gut microbiome have no effect on the peripheral serotonin. This may be due to the different effects of gut microbiome on serotonin in different regions of the body. Li et al. found that probiotic treatment reduced colonic serotonin and increased serotonin in frontal cortex and hippocampus, while there was no difference in peripheral serotonin [49], which is consistent with our results. One possible mechanism driving the neurobiological response and behavioral consequences of depression is glucocorticoid signaling induced by chronic stress. Chronic stress and glucocorticoid signaling can induce dendritic atrophy and functional changes in microglia which contribute to the deficits of behavior and cognition [50, 51]. Our results showed that rifaximin prevented the glucocorticoid elevation caused by CUMS. However, based on the results of our study, we cannot determine whether the gut microbiome directly affects the glucocorticoid, or whether the decrease of peripheral glucocorticoid is the result of stress relief in rats.

Morphologically, amoeba-like changes occurred in microglia, accompanied by an increase in volume and decrease in surface area, and increased levels of IL-1β and TNF-α were detected in the DG area of hippocampus, which suggests that CUMS enhanced the pro-inflammatory function of microglia.

We also found that CUMS enhanced the phagocytosis of microglia. Rifaximin heightened the anti-inflammatory function and normalize the phagocytosis of microglia. These results suggest that rifaximin may affect neuroinflammation in the DG by changing the functional state of microglia. We further found that butyric acid, which was affected by rifaximin, was negatively correlated with the content of TNF-α and IL-1ra and positively correlated with the content of IL-10 and IL-1β in DG, suggesting that butyric acid may be a key mediator of rifaximin in regulating the microglial function. Although growing evidence suggests that sodium butyrate(SB) has the ability to change the inflammatory phenotype of microglia [52], most researchers have used super-physiological doses of SB, which makes the reliability of the conclusion questionable. On the basis of the results of short-chain fatty acid determination, we explored whether a physiological dose of SB (0.3 μM) under the influence of rifaximin could inhibit the LPS induced activation of microglia in vitro. The results show that SB enhanced the anti-inflammatory function of microglia and prevented the phagocytosis of microglia induced by LPS. Combined with the results in vivo, these findings support the hypothesis that butyrate is the mediator of rifaximin regulating microglia function. The mechanism of SB regulating microglial function is not clear, which is one of our future research goals.

Adolescence is a vulnerable time for the onset of depression. Growing evidences demonstrate that chronic stress can reduce the number of newborn neurons and cause structural abnormalities and functional deficits [53, 54]. Our results show that the numbers of newborn and immature neurons in the DG of CUMS rats were significantly reduced, and the levels of TNF-α and IL-1β were increased. Neuroinflammation induced by microglial activation seems to inhibit neurogenesis in the DG, which is considered an indicator of depression [55]. The migration distance of immature neurons decreased in CUMS-treated rats, which disturbed the normal function of the hippocampal circuitry. Synapses participate in the formation of learning and memory through electrochemical signal transduction within highly specialized neural infrastructure [56]. It is generally believed that mushroom spines are responsible for the formation and long-term maintenance of memory [57]. CUMS reduced the density of mushroom spines. These results may explain the impairment of learning and memory behavior. Rifaximin prevented the decrease of newborn neurons and the deficient migration of immature neurons induced by CUMS. Rifaximin also increased the density of mushroom spines, which may be related to the regulation of microglia phagocytosis to some extent. These results suggest that rifaximin prevented the neurodevelopmental deficits caused by CUMS, which behaviorally reflected as the higher crossing frequency and longer residence time in the target quadrant in MWM. The mechanism of rifaximin improving neurodevelopmental deficits is not clear. Based on our experimental results, we speculate that there are the following possibilities: (1) Rifaximin may improve neurodevelopmental deficits by regulating the inflammatory function and phagocytosis of microglia via butyrate. (2) Rifaximin regulates neuronal proliferation and synaptic plasticity through butyrate, as the evidences showed that butyrate reverses maternal diet-induced neurocognitive deficits in offspring [58] and promote proliferation of human neural progenitor cells [59]. We will further verify the correctness of these assumptions in future work. More work is needed in the future to verify the correctness of these assumptions.

## Conclusions

In conclusion, CUMS can lead to changes in the microglial morphology and the secretion of inflammatory factors, resulting in the dysfunction of NPC proliferation and differentiation in the DG and leading to cognitive impairment and depressive-like behavior. The use of rifaximin, a non-absorbable antibiotic, can prevent the depressive-like behavior and cognitive impairment, which may be related to its ability to regulate inflammatory function of microglia by butyrate, a metabolite of gut microbiota, and protect against the neurodevelopmental deficits. These results demonstrate that gut microbiota and their SCFA metabolites play important roles in chronic stress disorder. Therefore, methods that modulate the gut microbiome and its metabolites may be a promising treatment strategy for depression.

## Supplementary Information

**Additional file 1: Figure S1.** The learning ability and spatial memory of rats. (A) Escape latency. (B) Duration in the target quadrant. (C) Entries in the target platform. (D) Movement trajectory. *P<0.05, **P<0.01, ***P<0.001 vs. the CON group; ^#^P<0.05, ^##^P<0.01, ^###^P<0.001 vs. the CUMS group.

**Additional file 2: Figure S2.** The integrity of intestinal mucosa. (A) The results of hematoxylin-eosin staining(HE). (B) Immuno-fluorescence for ZO-1(red), Claudin-1(Green) and DAPI(Blue) in colon. (C) The median fluorescence intensity(MFI) of Claudin-1. (D) The median fluorescence intensity(MFI) of ZO-1. *P<0.05, **P<0.01, ***P<0.001 vs. the CON group; ^#^P<0.05, ^##^P<0.01, ^###^P<0.001 vs. the CUMS group.

**Additional file 3: Figure S3.** The level of serotonin and glucocorticoid in plasma. (A) The level of peripheral serotonin. (B) The level of peripheral glucocorticoid. *P<0.05, **P<0.01, ***P<0.001 vs. the CON group; ^#^P<0.05, ^##^P<0.01, ^###^P<0.001 vs. the CUMS group.

**Additional file 4: Figure S4.** The morphologic analyse and the inflammatory cytokines of microglia. (A) The soma volume of microglia. (B) Immunofluorescence for Iba-1 (red) and CD68(Green). (C) Immunofluorescence for Iba-1 (red) and PSD-95(Green). (D) The median fluorescence intensity(MFI) of PSD95 in microglia. (E) The correlation between butyric acid and TNF-α, IL-1β. (I) The correlation between butyric acid and IL-10, IL-1ra.*P<0.05, **P<0.01, ***P<0.001 vs. the CON group; ^#^P<0.05, ^##^P<0.01, ^###^P<0.001 vs. the CUMS group.

**Additional file 5: Figure S5.** Changes in dendritic spines in DG. (A) Golgi staining. (B) The total number of dendritic spines. (C) The number of mushroom spines. *P<0.05, **P<0.01, ***P<0.001 vs. the CON group; ^#^P<0.05, ^##^P<0.01, ^###^P<0.001 vs. the LPS group.

## Data Availability

The data sets used and/or analyzed during the current study are available from the corresponding author on reasonable request.

## Abbreviations

CUMS: Chronic unpredictable mild stress
DAPI: 4,6-Diamidino-2-phenylindole
DCX: Doublecortin
DG: Dentate gyrus
GCL: Granule cell layer
ELISA: Enzyme-linked immunosorbent assay
HE: Hematoxylin and eosin staining
IFN-γ: Interferon-γ
IL-1β: Interleukin-1β
IL-1ra: Interleukin-1ra
IL-10: Interleukin-10
IL-4: Interleukin-10
LPS: Lipopolysaccharide
MDD: Major depressive disorder
MFI: Median fluorescence intensity
MWM: Morris water maze
NeuN: Neuronal nuclei
NPC: Neural progenitor cell
OFT: Open field test
OTUs: Operational taxonomic units
PC1: First principal component
PCoA: Principal coordinate analysis
PBS: Phosphate buffer saline
PFA: Paraformaldehyde
SB: Sodium butyrate
SCFA: Short-chain fatty acids
SGZ: Subgranular zone
SPT: Sucrose preference test
TGF-β: Transforming growth factor-β
TNF-α: Tumor necrosis factor-α
