# Supplementary material for: Rifaximin-mediated gut microbiota regulation modulates the function of microglia and protects against CUMS-induced depression-like behaviors in adolescent rat
Source: J Neuroinflammation. 2021 Nov 4;18:254. doi: 10.1186/s12974-021-02303-y (PMC8567657; doi:10.1186/s12974-021-02303-y)
Supplement: Supplementary file 1 — Additional file 1: Figure S1. The learning ability and spatial memory of rats. (A) Escape latency. (B) Duration in the target quadrant. (C) Entries in the target platform. (D) Movement trajectory. *P<0.05, **P<0.01, ***P<0.001 vs. the CON group; #P<0.05, ##P<0.01, ###P<0.001 vs. the CUMS group. [file 12974_2021_2303_MOESM1_ESM.pdf]

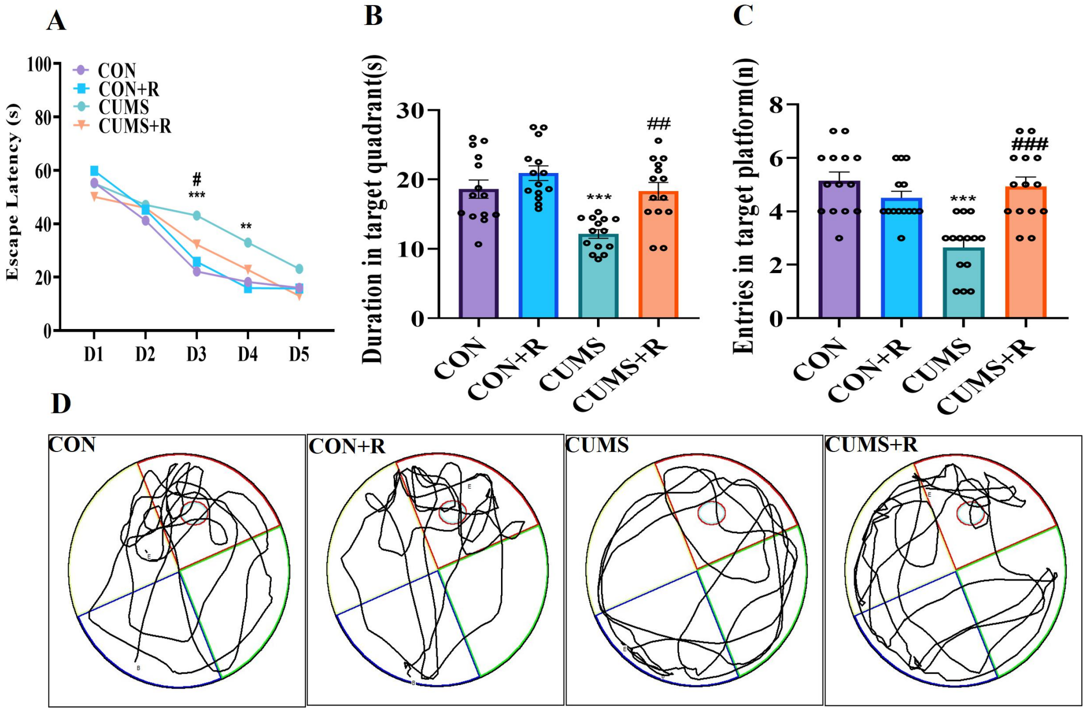

Supplemental Figure 1. The learning ability and spatial memory of rats. (A) Escape latency. (B) Duration in the target quadrant. (C) Entries in the target platform. (D) Movement trajectory. $^{*}P<0.05$, $^{**}P<0.01$, $^{***}P<0.001$ vs. the CON group; $^{\#}P<0.05$, $^{\#\#}P<0.01$, $^{\#\#\#}P<0.001$ vs. the CUMS group.
