# Supplementary material for: Rifaximin-mediated gut microbiota regulation modulates the function of microglia and protects against CUMS-induced depression-like behaviors in adolescent rat
Source: J Neuroinflammation. 2021 Nov 4;18:254. doi: 10.1186/s12974-021-02303-y (PMC8567657; doi:10.1186/s12974-021-02303-y)
Supplement: Supplementary file 2 — Additional file 2: Figure S2. The integrity of intestinal mucosa. (A) The results of hematoxylin-eosin staining(HE). (B) Immuno-fluorescence for ZO-1(red), Claudin-1(Green) and DAPI(Blue) in colon. (C) The median fluorescence intensity(MFI) of Claudin-1. (D) The median fluorescence intensity(MFI) of ZO-1. *P<0.05, **P<0.01, ***P<0.001 vs. the CON group; #P<0.05, ##P<0.01, ###P<0.001 vs. the CUMS group. [file 12974_2021_2303_MOESM2_ESM.pdf]

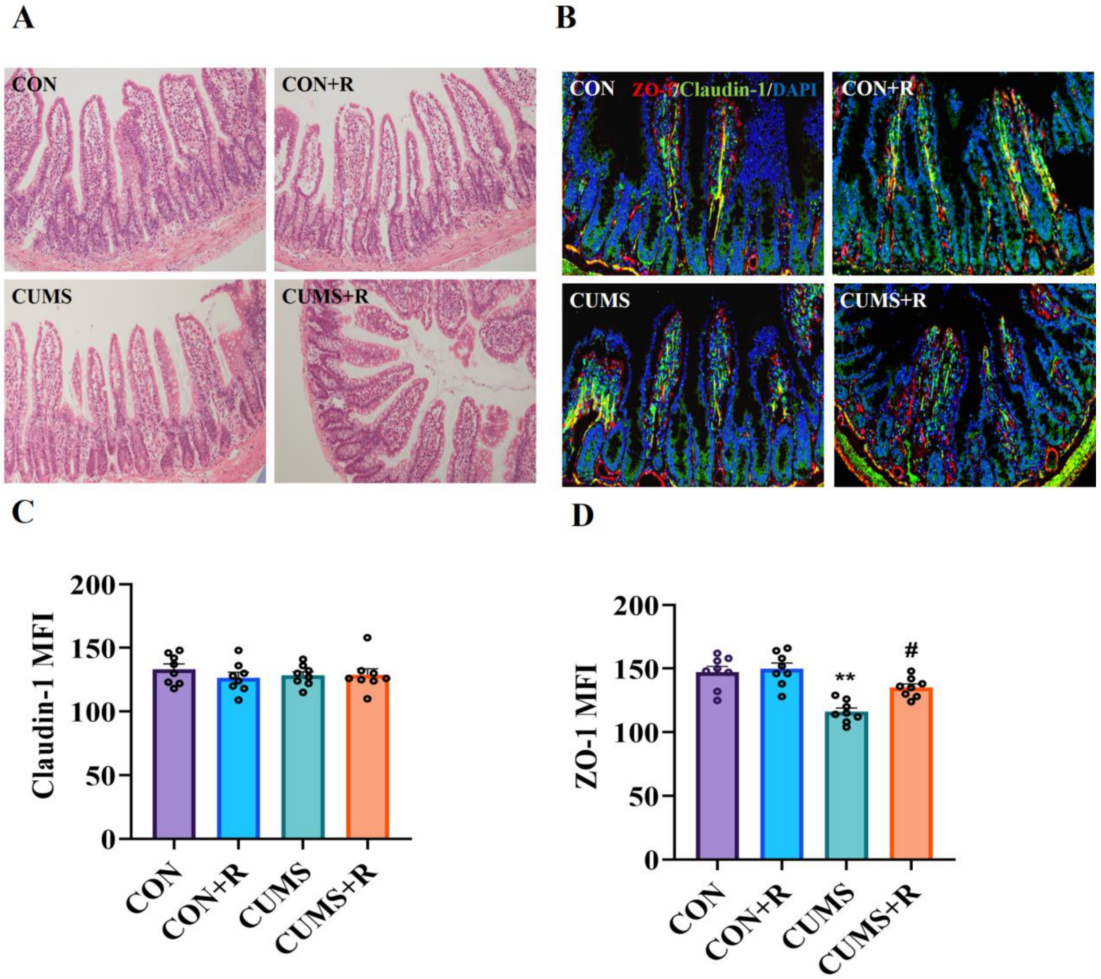

Supplemental Figure 2. The integrity of intestinal mucosa. (A) The results of hematoxylin-eosin staining(HE). (B) Immuno-fluorescence for ZO-1(red), Claudin-1(Green) and DAPI(Blue) in colon. (C) The median fluorescence intensity(MFI) of Claudin-1. (D) The median fluorescence intensity(MFI) of ZO-1. *P<0.05, **P<0.01, ***P<0.001 vs. the CON group; $^{\#}$P<0.05, $^{\#\#}$P<0.01, $^{\#\#\#}$P<0.001 vs. the CUMS group.
