# Supplementary material for: Rifaximin-mediated gut microbiota regulation modulates the function of microglia and protects against CUMS-induced depression-like behaviors in adolescent rat
Source: J Neuroinflammation. 2021 Nov 4;18:254. doi: 10.1186/s12974-021-02303-y (PMC8567657; doi:10.1186/s12974-021-02303-y)
Supplement: Supplementary file 4 — Additional file 4: Figure S4. The morphologic analyse and the inflammatory cytokines of microglia. (A) The soma volume of microglia. (B) Immunofluorescence for Iba-1 (red) and CD68(Green). (C) Immunofluorescence for Iba-1 (red) and PSD-95(Green). (D) The median fluorescence intensity(MFI) of PSD95 in microglia. (E) The correlation between butyric acid and TNF-α, IL-1β. (I) The correlation between butyric acid and IL-10, IL-1ra.*P<0.05, **P<0.01, ***P<0.001 vs. the CON group; #P<0.05, ##P<0.01, ###P<0.001 vs. the CUMS group. [file 12974_2021_2303_MOESM4_ESM.pdf]

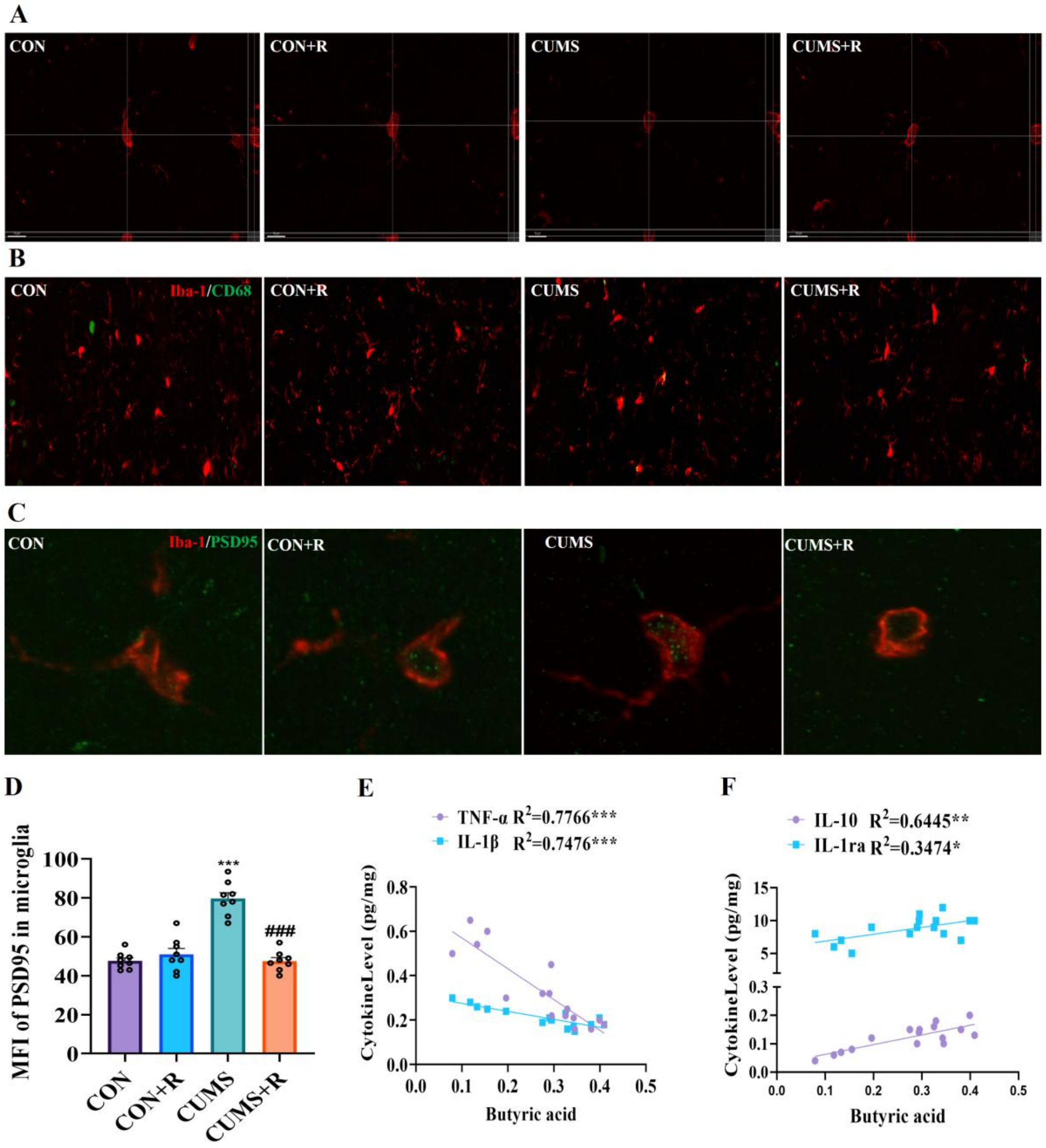

Supplemental Figure 4. The morphologic analyse and the inflammatory cytokines of microglia. (A) The soma volume of microglia. (B) Immunofluorescence for Iba-1 (red) and CD68(Green). (C) Immunofluorescence for Iba-1 (red) and PSD-95(Green). (D) The median fluorescence intensity(MFI) of PSD95 in microglia. (E) The correlation between butyric acid and TNF-α, IL-1β. (I) The correlation between butyric acid and IL-10, IL-1ra.$^{*}P<0.05$, $^{**}P<0.01$, $^{***}P<0.001$ vs. the CON group; $^{\#}P<0.05$, $^{\#\#}P<0.01$, $^{\#\#\#}P<0.001$ vs. the CUMS group.
